# Risk factors of cervical cancer after a negative cytological diagnosis in Polish cervical cancer screening programme

**DOI:** 10.1002/cam4.3857

**Published:** 2021-05-02

**Authors:** Anna Macios, Joanna Didkowska, Urszula Wojciechowska, Katarzyna Komerska, Patrycja Glińska, Michał F. Kamiński, Andrzej Nowakowski

**Affiliations:** ^1^ Department of Cancer Prevention Maria Sklodowska‐Curie National Research Institute of Oncology Warsaw Poland; ^2^ Department of Gastroenterology, Hepatology and Clinical Oncology Centre of Postgraduate Medical Education Warsaw Poland; ^3^ Polish National Cancer Registry Maria Sklodowska‐Curie National Research Institute of Oncology Warsaw Poland; ^4^ Department of Oncological Gastroenterology Maria Sklodowska‐Curie National Research Institute of Oncology Warsaw Poland; ^5^ Department of Health Management and Health Economics University of Oslo Oslo Norway

**Keywords:** cancer prevention, cancer risk factors, screening, women's cancer

## Abstract

Risk factors of cervical cancer (CC) development are well investigated, however, those influencing the risk of a potential false negative cytology preceding diagnosis of an invasive CC are not. We have aimed to explore these factors according to the data from Organised Cervical Cancer Screening Programme (OCCSP) in Poland. A total of 2.36 million of Pap tests sampled in 2010–2012 within OCCSP were merged with the Polish National Cancer Registry to identify CC cases after abnormal cytology and after normal cytology within 3 years of screening. Of 1460 invasive CCs, 1025 were preceded by abnormal and 399 by normal cytology result. Multivariate logistic analysis indicated that the presence of microorganisms in the Pap (OR = 2.18, 95% CI 1.65–2.87), evaluation by smaller (below 9000 slides processed per year) laboratories (OR = 1.60, 95% CI 1.22–2.09) and non‐squamous histology of cancer increased the odds for a potential false negative result (OR = 3.39, 95% CI 2.37–4.85 for adenocarcinoma, OR = 1.99, 95% CI 1.11–3.55 for other types of carcinoma), whereas cervical ectropion, other macroscopic changes on the cervix and smoking decrease the odds for a potential false negative Pap test result preceding CC (OR = 0.61, 95% CI 0.45–0.82, OR = 0.41, 95% CI 0.25–0.67, OR = 0.60, 95% CI 0.46–0.78, respectively). Proper triage of women with microscopic signs of microorganisms in the Pap smear should be reconsidered and cytology should be assessed in laboratories processing over 9000 slides annually to decrease the odds for negative Pap test result in 2 years before CC diagnosis. Information on macroscopic changes on the cervix provided to cytomorphologist may reduce the risk of a potential false negative cytology result.

## Graphical abstract

Although aetiology of cervical cancer is well established, risk factors for missing advanced cervical neoplasia in screening vary. In our study, on over 2.36 million of Pap tests higher risk of a potential false negative smear was associated with a low laboratory workload, and thus, we propose a minimum benchmark. Additional risk factors of a negative cytology result before cervical cancer diagnosis were presence of microorganisms in the smear and non‐squamous histology of cancer. Ectropion, other macroscopic changes of the cervix and smoking decreased the risk.

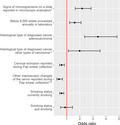

## 1 INTRODUCTION

Cytology‐based cervical cancer (CC) screening programmes are still the mainstay of secondary prevention of CC in many of developed countries around the world.^1^, ^2^ Although some of them have already switched to molecular testing for the presence of high‐risk human papillomavirus (hr‐HPV) in cervical samples as a more sensitive primary screening test, cytology still remains a component of the co‐test or an important part of triage protocols and is widely used in each country with HPV‐based screening implemented.^2^, ^3^, ^4^, ^5^

Despite the fact that cytological screening has successfully reduced the burden of CC in many countries worldwide,^6^, ^7^ Papanicolaou test is not a perfect screening tool due to its limited sensitivity and reproducibility.^8^ False negative Pap test results are common and pose a considerable risk of missing invasive and advanced preinvasive cervical neoplasia. Known factors responsible for false negative results include sampling errors, interpretation problems and true lack of abnormal cells in a properly collected sample related to the nature of the disease, for example, lesions developing high in the endocervical canal.^9^, ^10^ Some reports are known that long intervals and irregular screening may lead to false negative results.^11^, ^12^ However, other patient‐dependent and other factors affecting cytology results in women with occult invasive CC are still incompletely understood.

Organised Cervical Cancer Screening Programme (OCCSP) has been rolled out in 2006/2007 in Poland and is based on conventional cytology offered in 3‐year intervals free of‐charge to women aged 25–59 years.^13^ Pap smears are currently collected by gynaecologists and midwives in 1920 gynaecological clinics executing contracts with the National Health Fund (NHF) for gynaecological and obstetric care and in 119 of Family Medicine Centres. Taking Pap smear is a part of routine training during the medical specialty curriculum for gynaecologists. Midwives have to pass an exam to obtain certificate entitling to sampling within the OCCSP (such a certificate is not necessary for sampling aside the organised programme). Available data show no statistically significant differences in the rate of unsatisfactory for evaluation slides between midwives and gynaecologists. Slides are evaluated in 79 laboratories all over the country^14^ according to the modified Bethesda 2001 system.^15^, ^16^ Results of screening tests performed in 2010–2012 are presented in Table 1. In 2010–2012, 0.61% of slides were diagnosed as unsatisfactory for evaluation and about 2.65% were considered abnormal (include 2.48% of squamous and 0.10% of glandular lesions). High‐grade (atypical squamous cells, cannot exclude HSIL (ASC‐H), high‐grade squamous intraepithelial lesions (HSIL), squamous cell carcinoma (SCC), atypical glandular cells (AGC), adenocarcinoma in situ (ACIS), adenocarcinoma) and low‐grade (atypical squamous cells of undetermined significance (ASC‐US), low‐grade squamous intraepithelial lesions (LSIL)) abnormalities constituted 0.61% and 2.04% of all results, respectively. Of low‐grade abnormalities, 1.32% were ASC‐USs and 0.72% were LSILs. Among high‐grade abnormalities there were 0.19% ASC‐Hs, 0.29% HSILs, 0.02% SCCs, 0.10% AGCs and less than 0.01% of both adenocarcinomas and ACIS. Triage protocols incorporating a repeat cytology and a colposcopy with or without histological verification were set for women with abnormal results in 2008 by a group of experts from the Polish Gynaecological Society, the Polish Society of Pathologists and the Polish Society of Colposcopy and Uterine Cervix Pathology.^17^ For the ASC‐US diagnosis repeat smear in 6 months is recommended. LSIL may be triaged by colposcopy or repeat cytology in 6 months and it is to be decided by cytomorphologist. In the case of high‐grade lesions or second abnormal ASC‐US or LSIL result women are referred for colposcopy with biopsy. Quality assurance activities in the programme have been being developed for last 3 years and include audit of interval cancer cases which has been initiated in 2018. Since a detailed questionnaire is collected from each women participating in the OCCSP, which also includes results of vaginal speculum examination performed during Pap test collection, we have aimed to use this information in conjunction with data generated in invasive cancer audit to look for factors influencing the risk of potential false negative cytology reports preceding diagnosis of CC.

**TABLE 1:** Results of screening cytology examinations in Polish OCCSP in 2010–2012

Results of screening cytology in the Bethesda scale	Year of screening, *n* (%)			
	2010	2011	2012	2010–2012
Unsatisfactory for evaluation	5026 (0.63)	5076 (0.63)	4221 (0.55)	14,323 (0.61)
NILM	769,314 (96.65)	777,129 (96.72)	739,585 (96.87)	2,286,028 (96.75)
ASC‐US	10,996 (1.38)	10,445 (1.30)	9667 (1.27)	31,108 (1.32)
ASC‐H	1568 (0.20)	1455 (0.18)	1477 (0.19)	4500 (0.19)
LSIL	5795 (0.73)	5785 (0.72)	5499 (0.72)	17,079 (0.72)
HSIL	2313 (0.29)	2485 (0.31)	2114 (0.28)	6912 (0.29)
Squamous cell carcinoma	145 (0.02)	166 (0.02)	151 (0.02)	462 (0.02)
Total squamous lesions^a^	20,817 (2.55)	20,336 (2.47)	18,908 (2.42)	60,061 (2.48)
AGC	821 (0.10)	885 (0.11)	775 (0.10)	2481 (0.10)
ACIS	3 (0.00)	3 (0.00)	3 (0.00)	9 (0.00)
Adenocarcinoma	11 (0.00)	13 (0.00)	15 (0.00)	39 (0.00)
Total glandular lesions^b^	835 (0.10)	901 (0.11)	793 (0.10)	2529 (0.10)
Low‐grade lesions^c^	16,791 (2.11)	16,230 (2.02)	15,166 (1.99)	48,187 (2.04)
High‐grade lesions^d^	4861 (0.61)	5007 (0.62)	4535 (0.59)	14,403 (0.61)
Total abnormal results	21,652 (2.72)	21,237 (2.64)	19,701 (2.58)	62,590 (2.65)
Total	795,992 (100.00)	803,442 (100.00)	763,507 (100.00)	2,362,941 (100.00)

^a^ squamous lesions include ASC‐US, ASC‐H, LSIL, HSIL, squamous cell carcinoma.

^b^ glandular lesions include AGC, ACIS, adenocarcinoma.

^c^ low‐grade lesions include ASC‐US and LSIL.

^d^ high‐grade lesions include ASC‐H, HSIL, squamous cell carcinoma, AGC, ACIS, adenocarcinoma.

## 2 MATERIALS AND METHODS

We have analysed data from the screening database called SIMP (Polish: System Informatyczny Monitorowania Profilaktyki, IT System for Prevention Monitoring). Starting of 2007 SIMP recorded all procedures performed within the OCCSP. Healthcare providers can check there whether woman is eligible for screening. Next, data from routine questionnaire including personal information and data on gynaecological examination by smear‐collecting person is entered. Full information of results of both basic screening test and triage procedures are put into the SIMP. At each step of screening process medical staff is the only eligible for entering data and NHF is responsible for the database management. Opportunistic screening is not reported in SIMP.

We have retrieved data on all Pap smears collected in 2010–2012 within OCCSP. This time period covered the first round after the first fully registered round of screening in 2007–2009. The next round 2013–2015 would be eligible to be verified in Polish National Cancer Registry (NCR) in 2021 since 3 years of follow‐up and 2 years of delay in registration in NCR is needed to catch interval cancers. Each woman participating in OCCSP was sampled with conventional smear and slides were evaluated according to the modified Bethesda 2001 terminology.^13^ Data on women with cytological results obtained in the OCCSP were then linked with Polish NCR to identify CC cases diagnosed in those women within the screening interval of 36 months. NCR collects invasive carcinoma diagnoses; partial data are stored for preinvasive histology of lesions detected within OCCSP without a central database. Besides, according to European Guidelines, only fully invasive cancers should be included into the interval cancer audit. Database was checked and doubtful records were excluded from analysis (cytologies sampled within OCCSP after CC diagnosis or after date of death reported by NCR; women with date of death reported by NCR former to date of CC diagnosis; women above screening age). Each woman was attributed to her last slide. Smears sampled from women who were subsequently diagnosed with an invasive CC in up to 36 months since Pap test were considered as potential false negatives if the cytological diagnosis was *no intraepithelial lesion or malignancy* (NILM) or true positive in case of ASC‐US and more severe diagnoses. Data on histology of cancers were obtained from NCR, according to the International Classification of Diseases for Oncology (ICD‐O‐3).^19^

### 2.1 Collection of data on possible risk factors of negative cytology results in women with subsequent diagnosis of invasive CC

Cancer cases were divided into two groups according to the depth of cancer invasion: microinvasive and invasive carcinomas.^19^ Only fully invasive cases were included into the further analysis^18^ [p.166]. Each of them was then assigned to one of three following groups, according to the ICD‐O‐3 coding: squamous cell carcinoma, adenocarcinoma and other type of carcinoma which included: carcinoma, not otherwise specified (NOS); undifferentiated carcinoma, NOS; anaplastic carcinoma, NOS; small cell carcinoma; neuroendocrine carcinoma, NOS; leiomyosarcoma, NOS; adenosarcoma and carcinosarcoma.

Declarative data from a mandatory questionnaire obtained from each woman participating in the screening and entered into SIMP before sampling, selected for analysis included: (1) age at Pap smear collection, (2) education level (basic, secondary, higher), (3) parity (0, 1, 2, 3, 4 or more labours), (4) smoking status (non‐smoker, current, former), (5) hormone replacement therapy use (no/yes), (6) oral contraceptive use (no/yes), (7) intrauterine device presence (no/yes). Reference levels for subsequent analysis were given in the brackets in the first place.

Data on results of vaginal speculum examination during Pap test collection are recorded mandatorily by clinicians and the following were incorporated into our study, that is, (1) signs of colpo‐vaginitis at speculum examination, (2) cervical ectropion, (3) other macroscopic changes of the cervix defined as any of: papilloma, distortion, overgrowth, necrosis, polyp, tumour, infiltration or ulceration. Additional data on infections with microorganisms (*Trichomonas vaginalis*, *Candida albicans*, *Herpes simplex viruses*, *Bacterial vaginosis*, *Actinomyces*, *Chlamydia trachomatis* or other unspecific bacterial infection and changes in bacterial flora) are also routinely input by cytomorphologist into the SIMP and were included in the analysis.

To incorporate risk factors related to experience and workload of cytological laboratories, we used the number of smears evaluated by laboratory annually in 2010–2012 within the OCCSP. According to the European Guidelines laboratory should process at least 15,000 slides per year in an organised screening programme to maintain proper expertise.^18^ However, this number is based on experts’ opinion only and there are no sufficient evidences to support this view. In our sensitivity analysis, laboratories were divided into groups depending on the number of slides evaluated in a specific year, that is, into *below benchmark* and *above benchmark* groups and the benchmark was set at each 1000 between 2000 and 25,000 smears evaluated annually. The *above benchmark* group was the reference. Therefore, each slide was considered as processed by laboratory assessing at least or less than a fixed number slides per year.

Selection of variables for analysis of potential risk factors of negative cytology result before invasive CC occurrence was performed by an expert in cervical pathology and CC screening, based on data accessibility and literature.

Secondary purpose of the analysis was to estimate a proxy of sensitivity of cytology at the cut‐off of advanced cervical intraepithelial neoplasia (CIN) and invasive CC in age cohorts involved in screening (5‐year groups between 25 and 59‐year‐old) and to compare results between this groups. The study was approved by ethics committee of Centre of Postgraduate Medical Education (no. 126/PB/2019).

### 2.2 Statistical analysis

We compared CC cases diagnosed within 3 years after negative screening cytology result with those after positive cytological diagnosis using logistic regression. For each threshold of laboratories’ workload, we have selected the set of predictive variables from all potentially interesting ones using stepwise backward method with *p*‐value <0.1 considered as significant. Variables identified as significant by selection procedure were then incorporated into logistic regression model implemented in Stata 14.2^20^ software to estimate odds ratios (OR) for each benchmark. Best predictive model was chosen based on least deviance. Univariate analyses were carried to investigate impact of laboratories’ workload on occurrence of potential false negatives results.

We used percentage of CC cases diagnosed within 3 years after abnormal cytology results among both cases after normal and abnormal cytology to calculate a proxy of sensitivity of cytology at the cut‐off of advanced CIN and invasive carcinoma in OCCSP. Differences in a proxy of sensitivity stratified by age groups were tested using exact Fisher's test. Normality of variables was checked with Shapiro–Wilk test; differences between groups were tested with two‐sided tests χ^2^ test for ordinal variables and Fisher's exact test for binary variables. In the case of continuous non‐normally distributed variables, we used Mann–Whitney–Wilcoxon test for two groups and Kruskal–Wallis test for more than two groups. Significance level of <0.05 was established.

## 3 RESULTS

In 2010–2012, 2,362,941 Pap smears were collected; 198 of them (8.4/100,000 records) were rejected due to administrative errors and 2,362,743 records were left for analyses. Of 2,314,202 women who participated in screening, 4 were diagnosed with CC twice within at most 2 months and were linked with the former date of diagnosis. Overall 1514 CC cases (65.4/100,000 women participating in screening) were reported among eligible patients within 3 years after last cytological result collected in SIMP: 1460 cases (96.4%) were invasive cancers, 2 cases (0.1%) were microinvasive cancers and 52 (3.4%) of ICD‐O‐3 codes were missing. Of invasive cancers, 1025 (70.2%) were preceded by abnormal cytological diagnosis, 399 (27.3%) by normal diagnosis and 36 (2.5%) by smear inadequate for evaluation. We therefore identified 1025 cancer cases after abnormal and 399 cancer cases after normal cytology result. Laboratories evaluated mean number of 9724 slides annually. Characteristics of cancer cases by all available plausible factors which could modulate the ability of cytology to detect cervical neoplasia are gathered in Table 2. Histological type of diagnosed cancer, smoking status, presence of cervical ectropion during Pap test sampling, other macroscopic changes of the cervix reported while sampling, signs of colpo‐vaginitis at speculum examination, presence of microorganisms in the cytological sample and number of smears evaluated by a laboratory varied significantly between both cancer groups. Age, education level, parity, hormone replacement therapy use, oral contraceptive use and intrauterine device use were not significantly different between these groups.

**TABLE 2:** Basic analysis of factors influencing false negative and true positive cytological results in women with invasive cervical cancer diagnosed within 3 years after sampling in OCCSP in 2010–2012

Possible risk factors of false negative cytology result obtaining	Women with invasive cancer diagnosed within 3 years after		Univariate analysis
	False negative cytology result, *n* = 399	True positive cytology result, *n* = 1025	*p*‐value
Histological type of diagnosed cancer, *n* (%)			<0.001^b^
Squamous cell carcinoma	295 (73.93)	919 (89.66)	
Adenocarcinoma	83 (20.80)	72 (7.02)	
Other types of carcinoma^d^	21 (5.26)	34 (3.32)	
Age at examination, mean (SD)	47.11 (9.63)	47.22 (8.57)	0.563^a^
Education level, *n* (%)			0.164^b^
Basic	58 (16.02)	193 (20.21)	
Secondary	256 (70.72)	656 (68.69)	
Higher	48 (13.26)	106 (11.10)	
Parity, *n* (%)			0.061^b^
0	43 (10.78)	72 (7.02)	
1	97 (24.31)	233 (22.73)	
2	150 (37.59)	375 (36.59)	
3	62 (15.54)	196 (19.12)	
4 and more	47 (11.78)	149 (14.54)	
Smoking status, *n* (%)			<0.001^b^
Never smoked	233 (58.40)	469 (45.76)	
Quit smoking	139 (34.84)	495 (48.29)	
Currently smoking	27 (6.77)	61 (5.95)	
Hormone replacement therapy use, *n* (%)	14 (3.51)	36 (3.51)	1.000^c^
Oral contraceptives use (currently), *n* (%)	26 (6.52)	50 (4.88)	0.237^c^
Intrauterine device use (currently), *n* (%)	9 (2.26)	19 (1.85)	0.671^c^
Signs of colpo‐vaginitis reported during Pap smear collection, *n* (%)	13 (3.26)	65 (6.34)	0.020^c^
Cervical ectropion reported during Pap smear collection, *n* (%)	73 (18.30)	301 (29.37)	<0.001^c^
Other macroscopic changes of the cervix reported during Pap smear collection^e^, *n* (%)	21 (5.26)	143 (13.95)	<0.001^c^
Signs of microorganisms on a slide reported in macroscopic evaluation^f^, *n* (%)	132 (33.08)	195 (19.02)	<0.001^c^
Laboratories workload, *n* (%)			0.001^c^
Above 9000 slides processed annually	269 (67.42)	783 (76.39)	
Below 9000 slides processed annually	130 (32.58)	242 (23.31)	

^a^ Mann–Whitney–Wilcoxon test.

^b^ χ2 test.

^c^ Fisher's exact test.

^d^ include carcinoma, NOS; undifferentiated carcinoma, NOS; anaplastic carcinoma, NOS; small cell carcinoma; neuroendocrine carcinoma, NOS; leiomyosarcoma, NOS; adenosarcoma and carcinosarcoma.

^e^ include papilloma, distortion, overgrowth, necrosis, polyp, tumour, infiltration or ulceration.

^f^ include Trichomonas vaginalis, Candida albicans, Herpes simplex viruses, Bacterial vaginosis, Actinomyces, Chlamydia trachomatis or other unspecific bacterial infection and changes in bacterial flora.

### 3.1 Approximated sensitivity of cytology

**FIGURE 1:**
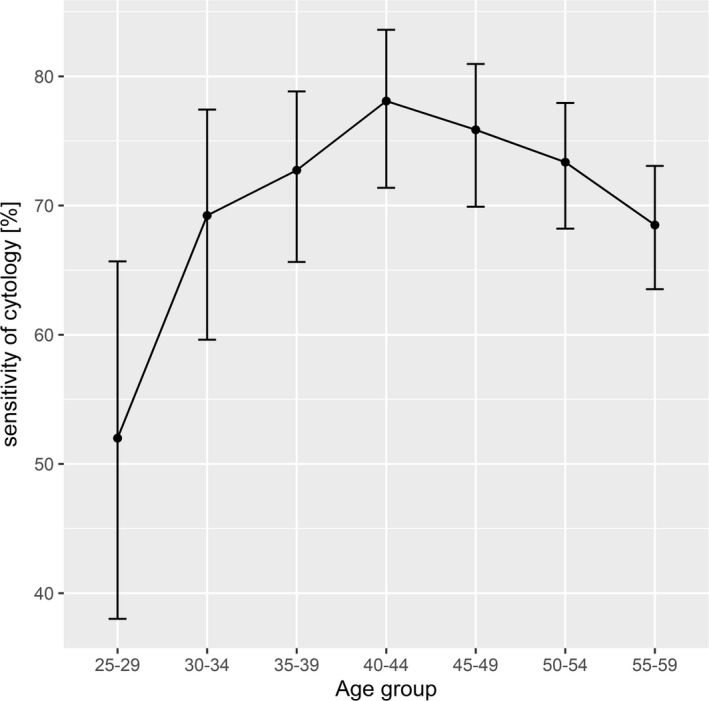
Approximated sensitivity of cytology at the level of advanced CIN and invasive CC in OCCSP in 2010–2012 by age group with 95% CI

A proxy of an overall sensitivity of cytology at the level of advanced CIN and invasive CC (see Figure 1) was calculated as 72.0% (95% CI 69.6%–74.3%). The lowest sensitivity of cytology (52.0%, 95% CI 38.0%–65.7%) was noted in youngest group of 25–29‐year‐olds. Approximate sensitivity was increasing up to age of 40–44 years, when the highest value of 78.1% (95% CI 71.4%–83.6%) was reached, and then declined to 68.5% (95% CI 63.5%–73.1%) in 55–59‐year‐old women. A proxy of sensitivity of cytology significantly depends on the age group (*p* = 0.007, χ^2^ test). Significant differences are reported between 25–29 and any other cohort (*p* = 0.049, 0.009, 0.001, 0.002, 0.004, 0.025, respectively) and between 40–44 and 55–59‐year‐old women (*p* = 0.020).

### 3.2 Independent risk factors of negative cytology result preceding CC occurrence

**FIGURE 2:**
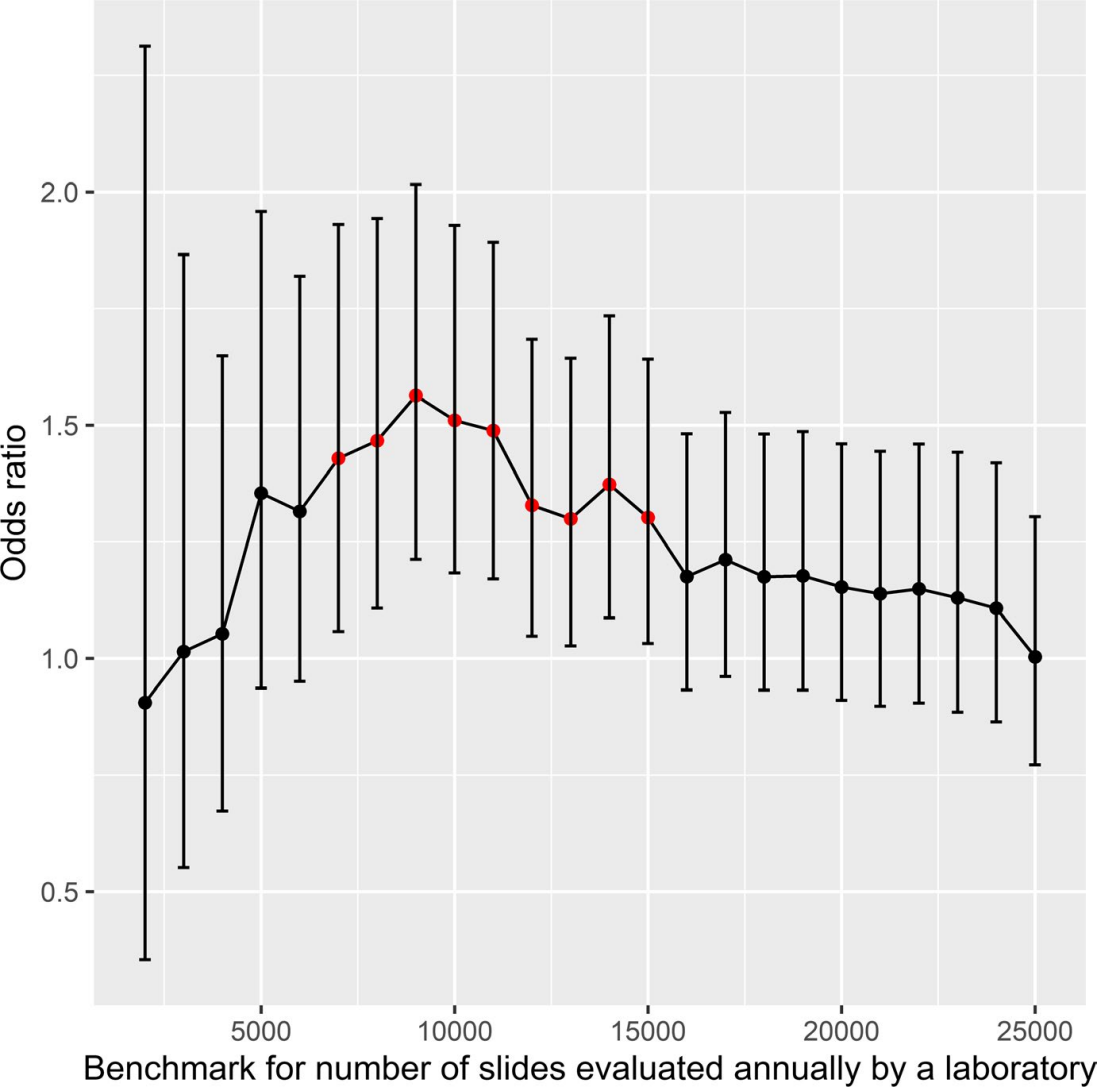
The ORs of interpreting the cytological slide of a subsequent CC case as a negative in OCCSP in 2010–2012, by the annual workload of the laboratory. Bars represent 95% CI for ORs. Significant results are marked with red dots

For each sensitivity analysis final multivariable logistic model included: *(1) histological type of cancer*, *(2) smoking status*, *(3) presence of cervical ectropion*, *(4) presence of microorganisms in the cytological sample*, *(5) other macroscopic changes of the cervix*, independently on the choice of the benchmark for laboratories’ workload and for those between 7000 and 15,000 slides processed annually also *(6) number of smears evaluated by the laboratory* was incorporated into the model. All variables included in multivariate models were found significantly influencing the risk for cytology to miss cervical neoplasia preceding diagnosis of invasive CC. Differences between results in sensitivity analyses were negligible for factors *(1) – (5)* (maximum difference in OR = 5%; data shown in the Table S1) and all results for these variables were significant. Reported ORs for laboratory workload peaks for benchmark of 9000 slides processed per year. Precise results of sensitivity analyses are collected in the Table S1. Univariate analysis results for each benchmark of laboratory workload are presented in the Figure 2. Peak in risk in univariate analysis coincides with the peak in multivariate analysis and is reported for laboratories processing below 9000 slides per year (OR = 1.56, 95% CI 1.21–2.02), which is similar to mean number of smears evaluated annually in 2010–2012 by a lab within OCCSP (*N* = 9764).

**FIGURE 3:**
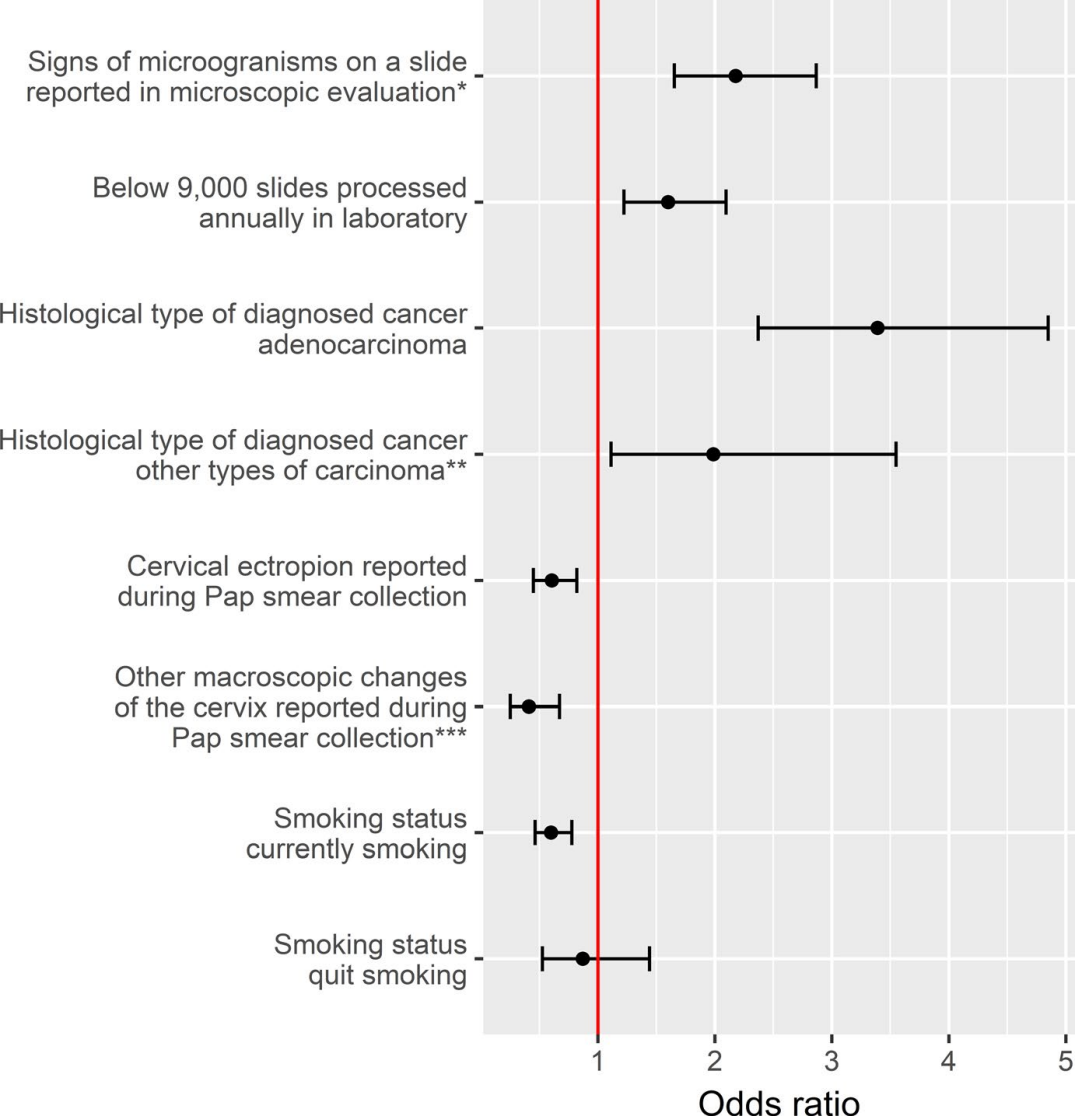
Multivariate logistic regression of factors influencing the interpretation of the cytological slide of a subsequent CC case as a negative in the OCCSP in 2010–2012–results of a multivariate logistic analysis. Bars represent 95% CI for ORs; ^*^include: *Trichomonas vaginalis*, *Candida albicans*, *Herpes simplex viruses*, *Bacterial vaginosis*, *Actinomyces*, *Chlamydia trachomatis* or other unspecific bacterial infection and changes in bacterial flora;^**^include: carcinoma, NOS; undifferentiated carcinoma, NOS; anaplastic carcinoma, NOS; small cell carcinoma; neuroendocrine carcinoma, NOS; leiomyosarcoma, NOS; adenosarcoma and carcinosarcoma; ^***^include: papilloma, distortion, overgrowth, necrosis, polyp, tumour, infiltration or ulceration

In the case of 9000 smears processed per year in OCCSP multivariable logistic regression model had least deviance and was selected as best predictive one. Results of this analysis are presented in the Figure 3. Presence of microorganisms elevated the risk of normal cytology result preceding cancer diagnosis (OR = 2.18, 95% CI 1.65–2.87). Odds of obtaining a potential false negative Pap result were higher for laboratories with at most 9000 slides processed annually comparing to those with more than 9000 Pap smears evaluated per year (OR = 1.60, 95% CI 1.22–1.71). Also risk of diagnosis of both adenocarcinoma and other non‐squamous types of carcinoma were higher among cases after normal than after abnormal screening result (OR = 3.39, 95% CI 2.37–4.85 and OR = 1.99, 95% CI 1.11–3.55, respectively). Both presence of cervical ectropion and other macroscopic changes of the cervix at Pap smear collection reported by clinicians decreased the odds of missing cervical neoplasia (OR = 0.61, 95% CI 0.45–0.82 and OR = 0.41, 95% CI 0.25–0.67, respectively). Smoking cigarettes reduced the odds of negatve cytology result preceding diagnosis of CC (OR = 0.60, 95% CI 0.46–0.78 for current smokers).

## 4 DISCUSSION

Our analysis on a large material of cytological samples collected throughout first full round after OCCSP roll‐out indicate that there are patient‐dependent and patient‐independent factors related to the risk of obtaining a negative result of a Pap smear preceding diagnosis of invasive CC within the standard 3‐year screening interval. Presence of microorganisms in the smear assessed as NILM and evaluation of the Pap smear in smaller laboratories provides less reassurance of the absence of cervical neoplasia. Also, the risk of diagnosis of non‐squamous invasive CC (both adenocarcinoma and other types) after a negative cytology result is higher than the risk of squamous cell CC. Factors decreasing the odds for a potential false negative screening cytology were ectropion, other macroscopic changes on cervix reported during Pap smear sampling and current smoking.

Significance of non‐HPV genital tract infections and inflammatory changes both diagnosed clinically and cytologically for CC screening has been widely discussed in literature ^21^, ^22^ and the issue of inflammatory and reactive changes in cervical cytology samples was the subject of updates in the Bethesda system. According to the Bethesda 2001 system and its 2014 modification,^15^, ^16^ slides with nonneoplastic cellular variations, reactive changes and microorganisms presence (i.e. *Trichomonas vaginalis*, *Candida*, *Bacterial vaginosis*, *Actinomyces*, *Herpes simplex virus*, *cytomegalovirus*) are included among normal smears, do not require triage and their reporting is optional.^18^, ^23^ The category of *atypical squamous cells of undetermined significance favour reactive* was eliminated from the previous Bethesda system terminology due to substantial referral rates for repeated sampling considered unnecessary. Several previous reports reviewed ^18^ indicate, however, that presence of inflammatory changes obscuring epithelial cells may be responsible for a various fraction of false negative cervical cytology reports.^24^ Some associations were found between microorganisms and HPV infection,^25^ and therefore, with higher risk of CC, that is, *Chlamydia trachomatis*,^26^, ^27^ *Herpes simplex virus*,^28^ *Bacterial vaginosis*.^29^ However, to our best knowledge, there are no studies indicating them as a reason of false negative cytological results. We have distinguished signs of colpo‐vaginitis visible during the speculum examination and infections diagnosed by cytomorphologist in microscopic evaluation and believe this would made our analysis more accurate. Unfortunately, no data on antibiotic treatment due to infections is collected in the SIMP and we cannot conclude about woman's further or previous diagnostic path. However, our analyses on a very representative population‐based material confirm higher risk of potential false negative reports of cytology when microorganisms occur in Pap smears and suggest reconsidering adequate form of triage for women with these results. Published studies point out lower rate of unsatisfactory for evaluation slides in the case of liquid‐based cytology use and more accurate result,^30^, ^31^, ^32^ however, this type of screening test was not available in Poland these days and still over 99% of screening tests are conventional smears. In 2019, we have initiated a randomised health services study comparing current standard of conventional Pap to a hr‐HPV‐DNA test with reflex LBC which should lead to implementation of hr‐HPV‐DNA‐based screening in Poland. After a full round of screening, we should be able to also seek for risk factors for false negative triage LBC in hr‐HPV‐based screening and compare the findings with our analysis.

Despite expert‐opinion‐based recommendations on the minimal number of annual Pap tests processed in the framework of organised screening by laboratory is set at 15,000^18^ [p.155], there has been insufficient data to establish an evidence‐based definite benchmark. Indeed, our data confirm that the risk of a negative cytological results preceding an interval CC is higher in smaller laboratories, irrespectively of chosen level of reference. Maintaining appropriate knowledge and skills in Pap smears assessment requires a sufficient workload which facilitates gaining experience by staff. Results of our study should raise a discussion about establishing adequate benchmark for minimal number of slides evaluated in laboratory within OCCSP. Our analyses show that 9000 slides evaluated annually best differentiate laboratories according to the level of potential false negative slides. Subsequently, laboratories with more than 9000 smears processed per year provide highest reassurance in terms of potential false negative cytology result to women participating in OCCSP. We could not consider whether and how slides evaluated outside the organised programme influence the expertise of staff. In Poland, the total amount of slides processed by laboratory outside the OCCSP is impossible to apprise since there was – and still there is – no central cytological database these days (2010–2012) and no reliable data source is available for opportunistic screening.

Adenocarcinoma and glandular intraepithelial lesions often escape cytological detection.^33^ This may be related to the location of the lesion deeper in the endocervical canal resulting in less efficacious sampling of glandular cells and difficulties in correct identification and assessment of abnormal glandular cells in the smear. Our results confirm previous reports ^18^, ^33^, ^34^, ^35^ and clearly indicate that risk of missing glandular neoplasia and other rare histological types of CC precursors in cytology‐based screening is significantly higher than missing squamous lesions. In our study, glandular and rare non‐squamous CCs constituted 14.7% of all cases (210/1424), but almost half of them (104/210, 49.5%) was not detected in screening.

We have found lower odds of obtaining a potential false negative cytology result in women presenting with cervical ectropion and other macroscopic changes of the cervix at speculum examination during Pap test collection. There may be several reasons to explain our findings. First, presence of cervical ectropion indicates presence of squamocolumnar junction and the transformation zone – where vast majority of neoplasia occurs – on the ectocervix and facilitates more accurate sampling of neoplastic cells. Second, macroscopic changes on the cervix may trigger more attention of the gynaecologists or midwives and provide higher quality of Pap smear collection. Third, information on macroscopic changes on the cervix in the questionnaire which serves as a referral letter to the cytology lab may result in more detailed assessment of the slides and higher sensitivity. A protective effect of cervical ectropion and other macroscopic cervical lesions and lower odds of interval CC in screened women we found in our analysis has not been studied thoroughly so far to our knowledge and requires further insight.

In our study, smoking seemed to lower the risk of interval CC. Some data ^36^, ^37^ indicate a positive relationship only between smoking and risk of squamous CC but no relationship with the risk of cervical adenocarcinoma. In our study, squamous cell carcinomas constituted 89.3% of considered invasive CC cases among smokers and 81.6% among non‐smokers (*p* < 0.001, Fisher's exact test). Since squamous cell carcinoma is more commonly properly diagnosed, therefore, risk of misdiagnosis among smokers may be lower than among non‐smokers. We postulate that possible biologic differences occur between CCs in smokers and non‐smokers which may be responsible for lower risk of interval CC in smokers. This finding certainly requires further elucidation.

Data from the USA in 2003, some time after the Bethesda scale introduction, pointed out 3.8% of ASC‐US and 2.1% of LSIL in conventional Pap test.^38^ Chinese data showed 2.33% of ASC‐US ^39^ in conventional smears taken in 2011–2015 and article from Italy indicated 1.9% of ASC‐US in organised screening in 2008–2010.^40^ In Poland, the ASC‐US rate has been stable as we have checked the database from 2010 to 2015 and is around 1%. Our unpublished analysis shows that the risk of cervical cancer is about 0.02% (95% CI 0.02%–0.02%) within 3 years after NILM cytological result and about 0.28% (95% CI 0.21%–0.35%) after ASC‐US. We speculate that low rate of ASC‐US follows from the type of training of Polish cytodiagnosticians since the rates are very consistent throughout the years.

Sensitivity of a test is one of the most important performance indicator in screening but has never been evaluated in OCCSP in Poland. We have estimated a proxy of sensitivity of cytology at the level of advanced CIN and invasive CC at 72.0% (95% CI 69.6%–74.3%) with lowest values in the youngest age group (25–29 years) and higher and generally stable rates for women 30–59 years of age (Figure 1). These findings confirm lower performance of cytology in young women suggested in some other studies.^35^ Since the risk of CC is very low in 25–29‐year‐old women in Poland,^41^ they are also more often expected to have regression of the precancerous lesions and are triaged and treated less aggressively. Nevertheless, some of these are true cancers or lesions progressing to invasive disease.

Published studies indicated highly varying sensitivity for cervical cytology, ranging from 30% to 87%.^42^, ^43^ In a population‐based study from England published in 2016, sensitivity of cytology at the second benchmark of abnormalities, which is similar to methodology in our study, reached almost 90% and was comparable throughout ages.^44^ United Kingdom CC screening programme has extensive quality assurance measures implemented. Substantially lower proxy of sensitivity of cytology in Poland indicates possible issues related to its performance, especially in young women and require further evaluation and corrective actions. A pilot randomised healthcare policy comparison of performance of hr‐HPV testing versus cytology has just been initiated in Poland ^45^ and its results should shed new light on the selection of the optimal screening test in our country^46^ [p.45]. We also performed a pilot review and are currently comprehensively re‐evaluating all potential false negative slides from the screening programme to find out specific reasons for lack of the detection of cervical neoplasia. These would include: *(1) true lack of cellular abnormalities*, *(2) inadequate quality of the smear* and *(3) misdiagnosis of an abnormal smear by cytomorphologists* and will be reassessed in the context of the multiple factors examined in the current analysis. This analysis should directly point out patient‐dependent and patient‐independent factors related to obtaining a negative cytological diagnosis preceding interval CCs.

A main limitation of our study is the lack of information on screening history of women. As recent researches indicate, proper adherence to screening intervals significantly lowers the risk of CC incidence ^47^, ^48^ and risk of squamous cell carcinoma increases with time since last negative screen.^49^ The differences between investigated groups may be therefore associated with no screening history or history of dysplasia.^50^ However, impact of regular screening on false negative cytology reports is not well investigated. In Poland, no central database with all performed cervical cytology results exists. NHF manages complete organised screening database since 2007 and also collects information on ICD‐9 (International Classification of Diseases) codes of procedures financed by health insurance. However, the procedures database is not reliable for the time before 2012 because there have been several changes in modes of coding procedures which impact reimbursement rates. Women with history of CC, cervical carcinoma in situ (CIS) and hysterectomy for any reasons are excluded from the programme, however, those with history of other cervical lesions or procedures are not. Women who participated in OCCSP have no cytology or final histology results recorded in the case of any procedures performed or lesions diagnosed outside the programme. Also, substantial part of opportunistic screening takes place in private healthcare without any registration. According to data collected by Central Statistical Office in 2014,^51^ over 75% of Polish women aged 20–59 declare to have undergone Pap testing within last 3 years, however, SIMP database reported only 22.2% target population coverage in 2012–2014.^14^ Bearing in mind all these factors, attempts to adjust our results for screening history could be a source of bias. Our results should therefore be considered as defining factors impacting the risk of a single false negative cytological result preceding diagnosis of invasive CC in all women eligible for screening.

Beside, screening history and HPV vaccination status is not included in the questionnaire routinely filled in before the screening examination. At the time of the OCCSP roll‐out, the coverage of opportunistic screening was unknown and was not included into the questionnaire. At present the questionnaire contains data on latest Pap test and its result, however, in 2010–2012, these data were not collected. Moreover, up to now HPV vaccination is not included in National Immunisation Programme in Poland and is not being reimbursed by the NHF. HPV vaccine is hard to reach even in private sector and is lacking in pharmacies and consequently the HPV vaccination coverage is low. Therefore, the impact of HPV immunisation on screening in Poland is minimal and may be negligible in our analysis.

In conclusion, we have identified the presence of microorganisms such as *Trichomonas vaginalis*, *Candida albicans*, *Herpes simplex viruses*, *Bacterial vaginosis*, *Actinomyces*, *Chlamydia trachomatis* or other unspecific bacterial infection and changes in bacterial flora on a slide, low workload in laboratory processing the slide and glandular neoplasia in final histological result as risk factors for obtaining a negative cytology result preceding diagnosis of invasive CC. On the contrary, presence of cervical ectropion, other macroscopic changes at speculum examination during Pap test collection and smoking cigarettes are factors decreasing risk of a potential false negative cytology result before occurrence of CCs. Therefore, we postulate further reconsideration of appropriate triage for women with NILM diagnosis but presence of microorganisms in the smear. Our findings should be verified in HPV‐based screening where cytological triage of HPV‐positive women is performed, especially in countries such as Turkey where conventional cytology is used.^3^ Also impact of laboratory workload on high‐quality screening programme should be investigated carefully to enable establishment of minimal number of slides processed by laboratory to receive contract with NHF for performing screening procedures within Polish and other countries settings. Our study points 9,000 slides evaluated annually as a potential benchmark. A proxy of sensitivity of cytology seems to be lower in young women aged 25–29 in OCCSP in Poland. Regardless of future switch to hr‐HPV examination as a primary test in Poland and many other countries, cytology may remain an important part of co‐test or triage protocols and reduction of potential false negative reports is essential to provide highest possible quality of preventive healthcare. Similar analyses would therefore be necessary to address cytology performance in a new landscape.

## CONFLICT OF INTERESTS

Authors declare no conflict of interests relevant to this article.

## Ethics Statement

The study was approved by the ethics committee of the Centre of Postgraduate Medical Education (126/PB/2019).

## Supporting information

Table S1

## Data Availability

The data that support the findings of this study are available from the corresponding author upon reasonable request.
